# A New Mass Burial of Cave Bears (Carnivora, Ursidae, *Ursus kanivetz*, Vereshchagin, 1973) from the Middle Urals

**DOI:** 10.1134/S0012496621030017

**Published:** 2021-06-25

**Authors:** P. A. Kosintsev, D. O. Gimranov, I. A. Lavrov, A. V. Kisagulov

**Affiliations:** 1 Institute of Plant and Animal Ecology, Ural Branch, Russian Academy of Sciences, 620144 Yekaterinburg, Russia; 2 Association of Ural Cavers, Kungur, Russia

**Keywords:** cave bear, *Ursus kanivetz*, Late Pleistocene, Ural, cave, taphonomy

## Abstract

Remains of a cave bear were studied from a new locality in the Prokoshev Cave in the Middle Urals (58°13´ N, 58°12´ E). Bones from all regions of the skeleton are present, bones are intact and without traces of human or animal activity. They all belong to the cave bear (*Ursus kanivetz* Vereshchagin, 1973). An AMS radiocarbon date of 53 375 ± 765 BP, IGAN_AMS_–8632, was obtained from an adult mandible. The bones belonged to at least 18 individuals, including 4 individuals aged about one year, 1 aged about two years, 1 aged about three years, and 12 individuals over four years of age. Three skulls belonged to males and seven skulls belonged to females. The analysis has shown that the taphonomic type of this locality is a “mass burial.” This is the first “mass burial” of the cave bear in the Urals, found in situ, untouched by humans.

Mass assemblages of carnivores not associated with human activity (the “mass burial” taphonomic type) are rare in the Late Pleistocene. Known examples include such assemblages of cave lions (*Panthera leo spelaea* Goldfuss, 1810) [1–3], wolves [3], the small cave bear (*U. savini* Andrews, 1922) [4, 5] and the brown bear (*U. arctos* L., 1758) [6–9]. The most common localities of this type are cave assemblages of the greater cave bear (*Ursus spelaeus* sensu lato) formed as a result of death during hibernation [10]. Studies of such localities yield unique information on the ecology and biology of these species.

Several caves with large numbers of cave bear bones are known from the Urals [11]. None of the taphocenoses in all of these caves remained intact; all of them had been altered by human activity prior to the study. The Prokoshev Cave, dealt with in this article, is a new cave bear “mass burial.” It is unique in preserving the assemblage in situ, because it had not been visited by humans prior to its discovery. The site thus offers an opportunity to study an undisturbed cave bear “mass burial.” The cave was named after N.A. Prokoshev, an archaeologist who studied caves along the Chusovaya River in the 1930s and died during the Second World War.

Prokoshev Cave is located in the Chusovaya River basin, Middle Urals (58°13´ N, 58°12´ E). It is karstic in origin and has a mixed structure [12]: a 100-m corridor ends in a cliff 8 m in depth and then passes into a large chamber with branches. The floor of the chamber is covered with limestone blocks and boulders with spaces between them filled with viscous brown loam. The “mass burial” is located in the large chamber. Bear bones lie on the surface of the floor and are partly or completely submerged in the loam. The bones are rather evenly distributed on the surface of the floor. No bones are anatomically connected. Chamber floor contains no traces of human stay.

The bones were collected from the cave floor, without extracting them from the loam. Sixty-four cave bear bones were collected (IPAE UD RAS, no. 2726): 13 skulls, 3 mandibles, 9 isolated teeth, 2 vertebrae, 5  ribs, 1 scapula, 4 pelvic bones, 3 humeri, 2 radii, 6 ulnae, 3 femora, 5 tibiae, 3 tarsals, 3 metapodials, and 2 phalanges. All the bones were intact, although several of the skulls and postcranial bones had some damage from water condensation. Bones had no bite or cut marks.

## *Age of the Fauna*

All bear bone remains have the same coloration and degree of fossilization. An adult mandible was used for AMS radiocarbon dating, yielding an age of 53 375 ± 765 BP, IGAN_AMS_–8632. This corresponds to the early Marine Isotope Stage 3 or the Bryansk (Moershoofd) Interstadial [13]. The degree of fossilization does not contradict this date.

### Taxonomic Identification

**Fig. 1.**
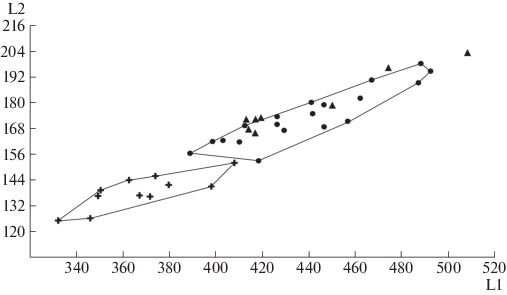
The ratio of the total length of the skull (L1) to the facial length of the skull (L2) of cave bears (in millimeters). (+) small cave bear; (●) large cave bear; (▲) bear skulls from the Prokoshev Cave.

The skulls have a steep curve in the forehead, which is typical of cave bears and untypical of brown bears [14]. Teeth (P4, M1, and M2) are without additional tubercles typical of the small cave bear. At the same time, P4 has a tubercle on the inner side of the metaconid, forming a transverse ridge, which also distinguishes the skulls from the Prokoshev Cave from those of the small cave bear [14]. Morphometric data show that the studied skulls are larger than the skulls of both the small cave bear and the Deninger bear, and correspond to the *U. kanivetz* (Fig. 1). Therefore, as judged by morphological and morphometric evidence, the skulls from the Prokoshev Cave belong to a cave bear (*U. kanivetz* Vereshchagin, 1973).

### Age Structure

The age structure of the assemblage was determined on the basis of a series of characters: the stage of tooth eruption, epiphyseal fusion, and long bone size [11, 15, 16]. Thirty-six bones belong to adults (older than four years), including 11 skulls, 2 lower mandibles, 9 isolated teeth, 1 vertebra, 2 ribs, 1 scapula, 2 pelvic bones, 2 humeri, 3 ulnae, 1 radius, 1 tibia, and 1 calcaneum from at least 12 individuals. One metapodial belongs to an approximately three-year-old individual. Bones belonging to two-year-old individuals include a mandible and a femur from one or two individuals. The bones belonging to one-year-old individuals include 2 skulls, 2 pelvic bones, a humerus, a radius, 2 ulnae, 2 femora, and 4 tibiae from at least four individuals. The bones belong to at least 18 individuals, including 12 adults. The sample includes all age groups except for the newborn group. The interval between the deaths of animals is about one year, which means that they died in the cave once a year, during hibernation.

### Sex Composition

**Fig. 2.**
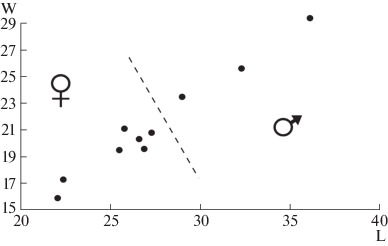
Ratio of the length of the upper canine alveolus (L) to the width of the upper canine alveolus (W) of cave bears from the Prokoshev Cave (millimeters).

Male and female cave bears are clearly distinguishable on the basis of the maximum size of upper and lower canines, with males having much larger canines [17]. On the basis of the sizes of the upper canine (C1) alveoli, the ratio between the maximum length (L) and maximum width (W) was plotted (Fig. 2). The skull sample was divided into two groups. The group with the larger alveoli consisted of males, and the group with smaller alveoli consisted of females. Three skulls are male and seven skulls are female. These results show that both males and females died in the cave. A considerably greater number of females died, compared to males. The predominance of females over males among the dead individuals was also found in several other caves in the Urals, including Ignatievskaya Cave, Tayn (Secrets) Cave, and Geologov 3 Cave [11].

The sample studied has a small size and a strongly biased composition. It contains mostly cranial bones, which reflects the specific composition of bone remains on the cave floor. After the death of the animals, their bones sank into the viscous unconsolidated sediments. The bones that stayed on the surface were the more bulky bones (skulls) and the bones that accidentally were not buried in sediments. Practically all bones are obviously contained in the soft sediments of the cave floor.

The sample contains remains of males and females of all age groups with the exception of newborns. The death of animals occurred once a year. There are bones from all parts of the skeleton; the bones are intact, without animal bite marks or human tool marks. This evidence shows that the locality is taphonomically a “mass burial” of animals that died during hibernation. The sample studied contains the remains of at least 18 individuals, which suggests that several dozen individuals died in the cave.
