# Erratum to: **A New Mass Burial of Cave Bears (Carnivora, Ursidae,*Ursus kanivetz*, Vereshchagin, 1973) from the Middle Urals**

**DOI:** 10.1134/S0012496621050112

**Published:** 2021-11-03

**Authors:** P. A. Kosintsev, D. O. Gimranov, I. A. Lavrov, A. V. Kisagulov

**Affiliations:** Institute of Plant and Animal Ecology, Ural Branch, Russian Academy of Sciences, 620144 Yekaterinburg, Russia

The article “A New Mass Burial of Cave Bears (Carnivora, Ursidae, *Ursus kanivetz*, Vereshchagin, 1973) from the Middle Urals” by P.A. Kosintsev, D.O. Gimranov, I.A. Lavrov, and A.V. Kisagulov, was originally published electronically in Springer-Link on June 25, 2021 without Open Access. After publication in volume 498, pages 79–81 the authors decided to make the article an Open Access publication. Therefore, the copyright of the article has been changed to © The Author(s) 2021 and the article is forthwith distributed under the terms of the Creative Commons Attribution 4.0 International License (http://creativecommons.org/licenses/by/4.0/, CC BY), which permits use, duplication, adaptation, distribution and reproduction of a work in any medium or format, as long as you cite the original author(s) and publication source, provide a link to the Creative Commons license, and indicate if changes were made.

